# Barriers to Oral Hygiene Self-Management Among Patients with Oral Cancer Treated with Radiotherapy: A Qualitative Study Using the COM-B Model

**DOI:** 10.3290/j.ohpd.c_1993

**Published:** 2025-06-05

**Authors:** Xing Gan, Lili Hou, Yuyang Li, Ying Yang, Xiaomei Zhao

**Affiliations:** a Xing Gan Researcher, School of Nursing, Chengdu University of Traditional Chinese Medicine, Chengdu 611137, Sichuan, China; Nursing Department, Afﬁliated Ninth People’s Hospital, Shanghai Jiao Tong University School of Medicine, Shanghai 200011, China. Writing – original draft, software, methodology, formal analysis, data curation, conceptualisation.; b Lili Hou Researcher, Nursing Department, Afﬁliated Ninth People’s Hospital, Shanghai Jiao Tong University School of Medicine, Shanghai 200011, China. Writing – review and editing, validation, supervision, conceptualisation.; c Yuyang Li Researcher, School of Nursing, Chengdu University of Traditional Chinese Medicine, Chengdu 611137, Sichuan, China; Nursing Department, Afﬁliated Ninth People’s Hospital, Shanghai Jiao Tong University School of Medicine, Shanghai 200011, China. Writing – original draft, supervision, data curation.; d Ying Yang Researcher, School of Nursing, Chengdu University of Traditional Chinese Medicine, Chengdu 611137, Sichuan, China; Nursing Department, Afﬁliated Ninth People’s Hospital, Shanghai Jiao Tong University School of Medicine, Shanghai 200011, China. Writing – original draft, supervision, data curation.; e Xiaomei Zhao Researcher, Nursing Department, Afﬁliated Ninth People’s Hospital, Shanghai Jiao Tong University School of Medicine, Shanghai 200011, China. Writing – original draft, data curation, conceptualisation.

**Keywords:** mouth neoplasms, oral hygiene, COM-B model, self-management

## Abstract

**Materials and Methods:**

Based on the capability, motivation, and opportunity-behaviour (COM-B) model, a qualitative study was conducted involving 18 patients with oral cancer receiving radiotherapy at a tertiary hospital from March to June 2024. Semi-structured face-to-face interviews were performed to investigate the oral hygiene self-management experience of patients. The results of the interviews were assessed by visual thematic analysis using NVivo 12 software. This article complied with the consolidated criteria for reporting qualitative research (COREQ).

**Results:**

Barriers to self-oral hygiene management in patients with oral cancer undergoing radiotherapy were grouped into three themes: (1) lack of capability (poor oral hygiene awareness, limited oral care knowledge, symptomatic distress), (2) lack of opportunity (financial burden, oral care product accessibility limitations, lack of specialised medical resources, inadequate family functioning), and (3) lack of motivation (low intrinsic motivation, heavy psychological pressure, lifestyle entrenchment).

**Conclusion:**

The capability and motivation of patients undergoing radiotherapy for oral cancer to manage their oral hygiene must be improved, along with external resources for oral hygiene management. The medical team needs to continuously improve guidance on self-oral hygiene management to meet individual needs.

Oral cancer is a type of head and neck cancer, including cancers of the tongue, gingiva, salivary glands, and other parts of the oral cavity.^16^ Approximately 4.84 per 100,000 people have oral and maxillofacial malignancies.^38^ Treatment of oral cancer usually involves a combination of strategies such as surgery, radiation therapy, and chemotherapy.^32^ Radiation therapy is a key treatment that effectively kills tumour cells and reduces tumour size. However, radiation therapy has serious side effects on oral tissues (including dry mouth, oral mucositis, and tooth loss).^42,44^ Salivary secretion decreases during radiation therapy, weakening the oral self-cleaning effect. Additionally, the risk of oral mucositis is high during radiation therapy, and the oral pain, ulceration, and discomfort caused by oral mucositis prevent patients from adhering to good oral hygiene management.^36,43^ The key to maintaining oral health is good oral hygiene; effective self-management helps alleviate symptoms and improve quality of life.^4^

Oral hygiene management helps minimise the amount of harmful bacteria in the mouth, improve oral hygiene, and keep the mouth clean, thereby reducing the load of harmful bacteria in oral secretions and the risk of developing infections.^6,11^ However, patients with oral cancer often struggle with self-oral hygiene management.^13^ Patients with head and neck cancer have poor oral hygiene compared to those with other cancers.^30^ Lim et al^22^ found low compliance with oral care among radiotherapy patients, with only 44 (14.9%) of 296 patients complying with oral hygiene requirements at post-radiotherapy follow-up. Factors such as pre-radiotherapy surgery, radiotherapy site, pain, and difficulty opening the mouth impact patient self-oral care; however, the specific mechanism of action remains unclear.^23,40^ Previous qualitative research has focused on the life experiences and challenges of patients with oral cancer or their adherence to oral hygiene behaviours; however, it lacks an adequate description of the factors influencing patients’ self-oral care during radiotherapy.^17,29^ To address this, we specifically adopted a patient-centred approach to capture qualitative insights into the challenges patients experience in their daily self-oral care. Focusing on the patient’s perspective allows a better understanding of the real-world complexities and emotional aspects of self-oral care that are often overlooked in clinical settings.

Using a theoretical framework to understand barriers to behaviour may be the most effective strategy, as suggested by researchers.^46^ Michie developed the capability, motivation, and opportunity-behaviour (COM-B) model in 2011, which includes three components: capability (C), opportunity (O), and motivation (M). Their interaction elicits behaviour (B),^28^ meaning that a person must have the mental and physical ability to perform the behaviour, the physical and social opportunity to engage in it, and the motivation to trigger it. The model is used to analyse the intrinsic mechanisms and barriers to self-oral hygiene behaviours in patients with oral cancer and to elucidate the mechanisms by which the barriers work. For example, even if patients have some oral care competence (C), they may still abandon oral hygiene behaviours (B) if they lack external support or social acceptance (O). Conversely, even when patients are highly motivated and competent (C/M), they may have difficulty adhering to effective oral care (B) because of external conditions (O).

Therefore, understanding how patients manage their oral hygiene, self-care strategies, specific problems faced, and psychological and emotional responses during radiotherapy can help develop personalised care interventions and improve self-care outcomes. This study used a qualitative approach based on the COM-B model to explore the personal experiences, perceptions, and barriers to daily self-care from the perspective of patients undergoing radiotherapy for oral cancer. We aimed to analyse common problems and factors influencing oral hygiene management behaviours in patients undergoing radiotherapy for oral cancer, providing a basis for developing targeted interventions.

## MATERIALS AND METHODS

### Design

Descriptive qualitative research methods were used. Semi-structured face-to-face interviews were used with patients undergoing radiotherapy for oral cancer. The methodology and reporting of this study followed the uniform criteria for reporting qualitative research (see Supplementary File 1).^45^

### Participants

Participants were recruited from the radiotherapy department of a tertiary care hospital in Shanghai, China. To ensure maximum variability, we used a purposive sampling and maximum variation strategy to capture a range of key factors: age, sex, education, occupation, tumour site, clinical stage, and treatment modality (operation type, total dose of irradiation, chemotherapy, and reconstruction).

Participants were selected based on the following: (1) age >18 years; (2) underwent radiotherapy for oral cancer and were in a stable condition; (3) were conscious and could express their views clearly; (4) provided informed consent and were willing to participate in this study. Patients with mental disorders or cognitive dysfunction were excluded from the study. To ensure heterogeneity of the study population and sample size saturation, we recruited participants based on data saturation criteria, and the final sample size for the interviews was determined based on the criteria of information saturation, information repetition in the interviews, and no new themes.^12^ As the interviews progressed, the information obtained from the interviews began to be repeated after the 18th participant, at which point the recruitment process stopped. The demographics of the participants are shown in Table 1.

**Table 1:** General information sheet of interviewees (N = 18)

NO	Age	Gender	Education	Occupation	Tumour site	Stage	Operation type	Total dose of irradiation, Gy	Chemotherapy	Reconstruction
P1	36	Male	College	Office clerk	Jaw	IV	Total maxillectomy	66	No	Regional flap
P2	66	Female	Primary school	Unemployed	Palatine	IV	Wide local excision	70	Yes	No
P3	46	Female	Middle school	Farmer	Jaw	II	Wide local excision	60	No	Regional flap
P4	48	Male	Primary school	Worker	Buccal	I	Wide local excision	66	No	Regional flap
P5	58	Male	High school	Unemployed	Gingiva	IV	Wide local excision	70	No	No
P6	68	Male	Primary school	Unemployed	Gingiva	I	Wide local excision	70	Yes	No
P7	65	Male	Middle school	Unemployed	Tongue	III	Partial glossectomy	66	No	Regional flap
P8	58	Male	Junior college	Farmer	Jaw	III	Wide local excision	60	No	Regional flap
P9	44	Male	College	Worker	Palatine	IV	Wide local excision	70	Yes	No
P10	61	Male	High school	Individual	Tongue	IV	Partial glossectomy	66	Yes	Regional flap
P11	59	Male	Junior college	Individual	Gingiva	IV	No	66	Yes	No
P12	55	Female	High school	Unemployed	Buccal	IV	Wide local excision	70	No	Regional flap
P13	64	Female	Middle school	Unemployed	Tongue	III	Hemi glossectomy	60	No	Regional flap
P14	47	Female	College	Unemployed	Buccal	II	No	70	No	No
P15	46	Male	College	Worker	Tongue	IV	Hemi glossectomy	60	Yes	Regional flap
P16	42	Female	Middle school	Office clerk	Mouth floor	IV	Wide local excision	66	No	No
P17	53	Male	High school	Programmer	Gingiva	III	Wide local excision	70	No	No
P18	56	Male	Middle school	Worker	Tongue	IV	Near-total glossectomy	60	Yes	Regional flap


### Theoretical Framework

The COM-B model suggests that individuals must have intrinsic motivation, capability, and opportunity (environmental factors) to complete the process of transforming undesirable behaviours into healthy behaviours. Capabilities refer to a person’s behavioural abilities, including physical and mental. Opportunities denote external factors that stimulate or lead to an individual’s behaviour, including physical and social opportunities. Motivation enables an individual’s behaviour to be elicited and includes spontaneous motivation and reflective motivation.^37^ The interview outline was initially developed based on the COM-B model and finalised after consultation with experts in oral oncology, dentistry, and nursing.

Semi-structured interview guide: 1) How do you clean your mouth? (capability); 2) Have you experienced any problems in cleaning your mouth? (capability); 3) Do you use any special oral care products (eg, mouthwash, toothpaste, mouthwash)? Did these products help? (Opportunity); 4) What are some of the reasons why you are able (or unable) to consistently clean your mouth properly? (Motivation).

### Data Collection

The results of the interviews were collected from March to June 2024. After obtaining written informed consent, semi-structured interviews were conducted by the same researcher depending on the time and physical condition of the respondents. The interviews took place in a quiet, undisturbed setting and followed the interview outline as the centre, with appropriate questioning, retelling, and summary. The entire process was recorded, and notes related to the interview were made. Each interview lasted 30–45 min. After the interviews, the researcher transcribed the collected information within 24 h. Data collection was stopped when no new information emerged.

### Ethical Considerations

This study was approved by the ethical review committee and conducted following the principles of the Declaration of Helsinki. The ethics committee of the Institutional Review Board of the Shanghai Ninth People’s Hospital, Shanghai Jiao Tong University School of Medicine (approval number: SH9H-2024-T66-1) provided ethical approval. All participants signed an informed consent form and were informed that they could refuse to answer any questions or withdraw from the study at any time.

### Data Analysis

The two researchers used Nvivo 12 to organise the text data of the semi-structured interviews based on the research objectives and research questions. They repeatedly read the interview data using the COM-B model, marked the important concepts and ideas, and open-coded the data. Next, they grouped similar and related codes into categories and subcategories, defined these groups, and identified the corresponding examples to explain the study’s findings. The coding information and results were discussed and adjusted with team members regularly until a consensus was reached. The results were shared with the respondents via telephone calls or WeChat to verify the authenticity of the content. Additionally, two randomly selected transcripts and portions of codes were reviewed by two independent researchers not involved in data collection and data analysis to validate the interpretation and refine the coding structure.

## RESULTS

Based on the COM-B model, the factors influencing oral hygiene management behaviours of patients undergoing radiotherapy for oral cancer were summarised as follows: lack of capability, opportunity, and motivation. These findings are detailed in Table 2.

**Table 2:** Themes and subthemes

Themes	Subthemes	Description
Lack of capability (C)	Weak awareness of oral hygiene	Participants believed that treating the disease was more important and neglected oral care
Lack of oral care knowledge	Participants lacked knowledge of oral care methods and products
Symptomatic distress	Participants were unable to adhere to daily oral care routines due to symptoms related to treatment (eg, difficulty opening mouth, pain)
Lack of opportunity (O)	Financial burden	Financial constraints limited participants’ ability to access preventive dental care and purchase oral care products
Oral care product accessibility limitations	Oral hygiene tools and products were not widely available in some areas
Lack of specialised medical resources	There was a lack of specialised oral care guidance
Inadequate family functioning	Incorrect oral hygiene habits were present in the home environment
Lack of motivation (M)	Decreased intrinsic motivation	Participants expressed dissatisfaction with the effectiveness of oral health management
Intense psychological pressure	Due to significant psychological stress, participants lacked strong motivation for oral care
Lifestyle entrenchment	Participants found it difficult to change longstanding poor oral hygiene behaviours


### Lack of Capability

#### Weak awareness of oral hygiene

The interviews revealed that some patients underestimated the importance of oral care measures and lacked awareness of behavioural changes. They considered treatment interventions to be more important and oral cleanliness to be less important. Some patients believed that brushing was only for maintaining oral comfort or self-image and did not affect their health. Consequently, they did not perform effective oral cleaning regularly or maintain adequate oral hygiene.

*I rinse my mouth when I feel like doing so or when my mouth feels uncomfortable. As I get older, the condition of my teeth worsens, but when I am sick and undergo radiotherapy, I pay more attention to my teeth, but sometimes I feel lazy. (P11)*

*I did not pay too much attention to this; there are times when I hear the doctor say that I need to pay attention to oral hygiene, but I think it is more important to treat the disease. (P16)*

#### Lack of oral care knowledge

Patients’ lack of understanding of effective oral care leads to poor oral hygiene management, such as uncertainty about choosing oral hygiene tools, toothpaste, and mouthwash, and uncertainty about the timing of oral care and the intensity of brushing the teeth. Some patients were not skilled in properly using oral care tools, which usually manifested as a lack of understanding of brushing techniques, flossing, and oral care steps.

*My daughter bought tooth rinsers for me, but I am not very good at using them. (P9)*

*I have to floss sometimes, but occasionally, I bleed when I use it, and my mouth is sick, so I am afraid to floss. (P14)*

*Most patients had undergone oral surgery and were afraid to touch the wounds, which caused them to keep their teeth unbrushed after the surgery. When I touch the wound while brushing my teeth, it hurts and bleeds; I am more worried about touching the wound. I am afraid that toothpaste will be bad for the postoperative wound. (P1)*

*I was afraid to open my mouth after the operation; how was I supposed to clean my mouth? (P15)*

#### Symptomatic distress

Patients with oral cancer undergoing radiotherapy may experience oral symptoms such as reduced mouth opening, dry mouth, pain, and altered senses of taste and smell, making it difficult to maintain routine oral care habits. Additionally, they may experience fatigue and malnutrition during radiotherapy, which can decrease their frequency of brushing and flossing.

*After radiotherapy, I feel that I cannot open my mouth, and there are ulcers inside, which are particularly painful. I do not dare to touch them at all; I want to brush my teeth when the situation is better, and now I just rinse my mouth with water or mouthwash. (P3)*

*I cannot even brush my teeth hard now because this side of my face is swollen, and it hurts when I do it. (P12)*

*After the operation, I feel especially tired and have no strength; I have to use a wheelchair when I come here; sometimes, I do not use a toothbrush; I just rinse my mouth with mouthwash. (P11)*

Cervical lymph node dissection is a crucial method in the comprehensive treatment of oral cancer. However, patients cannot lift their arms after the procedure, coupled with the effects of radiotherapy. Additionally, wounds from flaps taken from the arm can limit their ability to perform oral care.

*I also had surgery here (shoulder and neck). I cannot lift my arms, I cannot brush my teeth, my family helps me clean my mouth when they have time, but I cannot brush my teeth by myself. (P18)*

### Lack of Opportunity

#### Financial burden

Because of financial pressure, some patients may reduce their regular oral examination and treatment, impacting timely intervention for oral health issues. Some patients said they would not visit a clinic or hospital for dental cleaning unless necessary. Additionally, some could not afford necessary oral care products such as specialised toothpaste, mouthwash, and electric toothbrush.

*I heard from the doctor about getting my teeth cleaned, but ever since I had this tumour, I have been spending money. My son is not married yet and has plenty of money to spend. Feeling clean teeth did not have much effect; so I did not go. (P2)*

*I only use children’s toothbrushes; I have heard that electric toothbrushes and toothpaste with fluoride are better, but I prefer whichever is cheaper. (P6)*

#### Oral care product accessibility limitations

Patients from poor and remote areas were unaware of appropriate oral care tools and products and did not know how to choose them. Some patients reported forgetting to carry their oral care tools when travelling.

*I am a farmer. I have never seen what an electric toothbrush or flosser looks like. (P5)*

*It is just a bit of a hassle; you have to take your toothbrush and all that when you travel, and sometimes you forget. (P13)*

#### Lack of specialised medical resources

A lack of professional guidance on oral care and regular check-ups may prevent patients from accessing effective care programmes and advice on adjustments. Some patients expressed the need for professional guidance.

*I wanted to understand how to manage my mouth after the operation. I asked the doctors and nurses and they only said that I should use mouthwash and pay attention to oral hygiene, but I am not sure exactly what I have to do. I hope that there are professionals who can guide me. (P3)*

#### Inadequate family functioning

Support and assistance from family members encourage patients to adhere to long-term oral management. For example, P2 and P10 mentioned that family supervision helped them adhere to oral care. However, a lack of awareness of the importance of oral care in the family can affect patients’ care behaviour.

*In my family, we always brush our teeth at night. We do not brush our teeth during the day, my parents do that, and nobody uses mouthwash in the family. (P8)*

### Lack of Motivation

#### Decreased intrinsic motivation

During the interviews, we found that a discrepancy between the status quo and expectations can lead to a loss of motivation for oral care when patients experience complications from radiotherapy.

*I used to brush my teeth all the time, and I still got cancer. Now, after radiation therapy, I do not feel like brushing my teeth is useful because I still have ulcers. (P17)*

*I want to give up when I have to come in for radiotherapy every day. What is the point of cleaning my mouth; it is not going to make me better. (P4)*

#### Intense psychological pressure

Patients with oral cancer often face considerable psychological stress, which arises mainly from the disease, the treatment process, and its side effects. Long-term psychological stress makes patients feel tired or frustrated, which reduces their motivation to manage oral hygiene.

*When I think about my illness, I do not want to do anything; it does not make sense. (P7)*

*Since having this illness there are times when it feels like there is no hope in life, you do not want to do anything, and it feels like there is no point in cleaning your mouth. (P16)*

#### Lifestyle entrenchment

A lifestyle that neglects oral hygiene may prevent patients from changing existing care behaviours even when new oral health problems arise during radiotherapy.

*My doctor told me to keep up with my oral hygiene. I have never been in the habit of maintaining my oral hygiene before. I have never used mouthwash, and now it is difficult for me to use it for a long time. (P12)*

## DISCUSSION

This study applied the COM-B model to analyse barriers to oral hygiene self-management in patients undergoing radiotherapy for oral cancer. Analysis of the three core elements of the model (capability, opportunity, and motivation) revealed that their interactions strongly influence patients’ adherence to effective oral care. Therefore, oral hygiene interventions for patients undergoing radiotherapy for oral cancer should be multifaceted, focusing on enhancing patients’ knowledge and skills, improving their external environments, providing adequate resources and support, and motivating behaviours through psychological and social support.

Patients undergoing radiotherapy for oral cancer face physical and psychological challenges that directly affect their oral hygiene behaviours, with psychological competence playing an important role. Patients often neglect appropriate oral care measures or underestimate their importance because of limited knowledge about oral hygiene or misconceptions about radiotherapy’s side effects. Interventions should focus on strengthening oral health education, highlighting the risks of poor oral hygiene, and increasing their awareness of hygiene management.^2,25^ Additionally, patients should be taught oral self-examination methods to help them establish and maintain good oral hygiene habits.^27^ However, unilateral knowledge guidance by healthcare professionals may ignore patients’ teach-back, resulting in a limited understanding of health knowledge and an inability to form long-term effective self-management behaviours. Therefore, teach-back oral health education, which involves repeated teaching and feedback until patients master oral health knowledge and skills, may address these issues effectively.^41^

We found that patients lacked knowledge about oral care products and did not know how to choose them. Healthcare professionals can address this issue by demonstrating each item in the oral care kit and explaining its use and function to patients with oral cancer before radiation treatment, ensuring they use the products correctly. Cullen and his research team distributed oral care kits to patients with head and neck cancer at a radiation oncology medical centre.^9^ The kits contained essential oral care products, such as soft-bristled toothbrushes and specific toothpaste, along with a large supply of pre-packaged mouthwash. They found that the oral care kits helped patients effectively manage their oral health and improved their adherence to oral care.

The patient’s physical capability is limited during radiation therapy, which affects the execution of their oral hygiene. Surgery, as the main treatment, disrupts the normal tissue anatomy of the oral cavity.^7^ Radiation therapy affects saliva secretion, reduces osteoclast activity, and causes muscle fibrosis, resulting in complications such as pain in the oral cavity, difficulty opening the mouth, dry mouth, and swallowing disorders.^5^ Additionally, after radical cervical lymph node dissection, patients find it difficult to lift their arms, feel exhausted, and experience low energy during treatment, which may reduce their willingness and ability to clean their mouth. Gautam et al^14^ reported that the use of prophylactic low-intensity laser therapy decreases the incidence of severe oral mucositis, pain, and dysphagia. However, this method cannot be generalised to all patients with oral cancer and may increase their financial burden. Healthcare professionals should provide detailed, tailored oral hygiene instructions to patients undergoing radiation therapy to effectively address poor oral hygiene caused by the side effects of these treatments.^20^ We recommend that patients take different measures based on their circumstances. For example, patients with oral mucositis need to use a specified mouthwash, while those with clenched teeth should perform mouth-opening exercises. Patients who cannot care for themselves or are fatigued need caregiver assistance with oral hygiene. Flossing is discouraged in patients with severe neutropenia or thrombocytopenia undergoing chemotherapy.^36^

In the COM-B model, opportunity refers to the external environment in which an individual lives and the availability of resources. For patients undergoing radiotherapy for oral cancer, access to adequate resources and support is crucial for performing oral hygiene behaviours. Our study found that economic status was one of the factors that influenced patients’ self-oral care. The financial burden of purchasing oral care aids, regular visits to the dentist, and treatment costs led some patients to neglect the importance of oral care. Pateman et al^31^ similarly found that financial pressures can limit patients’ behaviour in seeking professional dental services and purchasing complementary preventive products. Dental insurance may help alleviate the financial burden on patients. According to data collected by the National Centre for Health Statistics’ 2019–2020 National Health Survey in the United States, people with dental insurance are more likely to obtain oral healthcare and use healthcare services.^35^ Expanding oral visit health insurance coverage is an important way to help patients strengthen their oral health management behaviours, such as including preventive dental care (check-ups, cleanings, fluoride treatments, extractions) in dental insurance,^21^ as well as more financial support that may lead more patients to consider more effective oral health products and private dental care services. However, expanding insurance coverage requires investing more resources, including funding, healthcare delivery system development, and insurance administration. Caution is needed to ensure the scientific basis and sustainability of the policy. In addition to social support, family support is one of the factors influencing patients to engage in oral cleaning behaviours. Family members play an important role in the self-management of patients’ oral health problems.^15^ Training family members in oral care and encouraging their active involvement can improve motivation and compliance with oral hygiene behaviours.^26^

We found that patients in remote areas face difficulties accessing professional oral care resources. In addition to poorer educational status and economic conditions, the use of oral care products is lower among patients in rural areas.^1^ Cultural background is important in shaping oral health behaviours and dental service acceptance among individuals and groups.^33^ The importance of oral hygiene and behavioural patterns vary across cultures. Different cultures’ beliefs, habits, social structures, and understanding of health may determine how much oral care is valued, performed, and adhered to by patients. An Austrian study found that many people do not visit the dentist until they have a noticeable problem (eg, toothache), having grown up in an environment where they received fewer or no professional dental cleanings.^3^ People in certain traditional societies and cultures are more inclined to use traditional oral hygiene methods to maintain oral health and may be less likely to be actively involved in oral health management.^10^ Oral care may be neglected, especially during cancer treatment, where the focus is primarily on survival and treatment outcomes.^34^ Cultural differences must be considered when developing oral health management strategies to provide patients with more effective and individualised support and interventions. Despite the need for specialised care, many patients with oral cancer do not receive adequate instruction on oral hygiene management during treatment. Oral health management can be integrated into the oncology treatment plan by incorporating regular professional dental care into follow-up care and assessing the patient’s oral hygiene status using a plaque score or plaque index.^18^ Providing personalised services to patients according to their cultural background and knowledge level can facilitate smooth cancer treatment. However, this requires adequate resource support, including the training and staffing of professionals, investment in materials and equipment, and coordination of individualised oral health management with oncology treatment protocols. Consideration is given to the potential increase in complexity of the treatment process, patient compliance, and cost-effectiveness. Ultimately, the decision to incorporate oral health management should be based on hospital resources, patient needs, and the individualised requirements of the treatment plan.

Motivation is the central driver of oral hygiene behaviours in patients undergoing radiotherapy for oral cancer, determining the execution of these behaviours. Patients’ intrinsic motivations, such as their perceptions of oral health and expectations of treatment outcomes, directly influence their willingness to take active oral care measures. Some patients indicated in interviews that they did not view oral cleaning behaviours as meaningful. Their previous lifestyles, characterised by neglect of oral hygiene, impeded their ability to make changes, thereby reducing their motivation to engage in oral care. For many patients, managing oral hygiene may not be a priority, especially when faced with more serious cancer treatment challenges.^8^ Healthcare professionals should recognise the difficulties patients with oral cancer face with oral hygiene after radiation therapy. Before radiation therapy, they should assess patients’ past oral health behaviours and correct their unhealthy oral hygiene behaviours and perceptions.

Additionally, patients’ lack of intrinsic motivation and high psychological stress can affect their long-term adherence to effective oral care. Fear of treatment, concerns about their condition, changes in appearance, and discomfort from radiation therapy (such as pain and oral discomfort) may lead to negative emotions, which may reduce their motivation for daily oral care. At this stage, providing emotional support and counselling is crucial to help patients manage negative emotions promptly and enhance their willingness to adhere to their oral care regimen. Manne et al^24^ found that self-management efficacy in patients with oral cancer was associated with psychological factors, and patients with a high level of depression could less manage their self-care. We recommend a multidisciplinary approach to managing patients’ oral health. Multidisciplinary collaboration is key to providing effective stress management and psychological interventions that help patients cope with the psychological challenges of the treatment process through holistic support.^19^ The model of multidisciplinary teamwork in cancer care has been validated as a key factor in improving the quality of care and patient satisfaction.^26^ The multidisciplinary healthcare team includes oncologists, oncology nurses, dentists, linguists, social workers, psychologists, and other allied health professionals. Patients can be supported with a full range of treatments and care and are helped to find and access oral healthcare resources to ensure that all aspects of their needs are met.^3^ During radiotherapy, the team must understand the patient’s psychological condition and needs, tailoring psychological interventions by assessing the patient’s specific situation (eg, mood, cognition, social support). For example, after each radiotherapy session, in addition to providing health education on oral hygiene management, short-term psychological counselling can be offered to patients. It helps patients alleviate anxiety and fear caused by treatment, enhances their confidence in self-care, and strengthens their motivation for oral self-care.

### Strengths and Limitations

This study contributes to the understanding of self-managed oral hygiene behaviours in patients undergoing radiation therapy for oral cancer. The use of the COM-B model allowed the results of this study to be mapped directly to behavioural analyses, facilitating the empirical and theoretically based construction of intervention programmes for behavioural change. However, this study has a few limitations. First, it was primarily based on theoretical analysis and lacked practical validation of intervention effects. Second, the participants were from the same region and shared similar ethnic and cultural backgrounds, limiting the generalisability of the findings. Therefore, multicentre studies are needed to enrich the results of this study.

### Implications for Future Research

We established a conceptual framework for oral health management behaviours in patients with oral cancer by conducting an experiential study on the barriers to oral health management in patients undergoing radiotherapy. Our findings provide a foundation for future research. Researchers should investigate the effect of medical staff on barriers to self-oral health management in patients with oral cancer from the perspective of medical staff, especially their experience of dealing with this problem and how to promote positive and effective oral health management among patients.

## CONCLUSION

Based on the COM-B model and in-depth interviews, this study analyses and summarises the obstacle factors of self-oral hygiene management behaviours in patients undergoing oral cancer radiotherapy. The causes of poor oral hygiene behaviours are complex, influenced not only by individual ability and motivation but also closely related to cultural background, socioeconomic conditions, psychology, and other factors. These obstacles lead to difficulties for patients during treatment, especially in the daily care of oral health, which seriously affects the effectiveness of oral health management. The study highlighted the need to strengthen oral health education, provide psychological support, improve the allocation of medical resources, increase professional support for oral health management, and offer personalised advice and treatment plans for patients. Future interventions should consider patients’ abilities, opportunities, and motivations, adopting a multidimensional strategy to improve oral health compliance, enhance overall health during radiotherapy, and promote treatment outcomes.

### Ethical Approval

This study was approved by the Ethical Review Committee of the Affiliated Ninth People’s Hospital, Shanghai Jiao Tong University School of Medicine.

### Declaration of Competing Interest

The authors declare that they have no known competing financial interests or personal relationships that could have appeared to influence the work reported in this paper.

**Supplementary File 1:** Consolidated criteria for reporting qualitative studies (COREQ): 32-item checklist

No.	Item	Guide questions/ description	Checklist of this study
Domain 1: Research team and reflexivity
Personal characteristics
1.	Interviewer/facilitator	Which author/s conducted the interview or focus group?	Gan-Xing, Yang-Ying
2.	Credentials	What were the researcher’s credentials? eg, PhD, MD	MD
3.	Occupation	What was their occupation at the time of the study?	Interviewers, data analysts, writers, and revisers of first drafts of the paper
4.	Gender	Was the researcher male or female?	All female
5.	Experience and training	What experience or training did the researcher have?	All the authors, including GX, HLL, YY and LYY, have received training in qualitative research methods as part of an ongoing Master’sproject
Relationship with participants
6.	Relationship established	Was a relationship established prior to study commencement?	No
7.	Participant knowledge of the interviewer	What did the participants know about the researcher? eg, personal goals, reasons for doing the research	Reasons for doing the research
8.	Interviewer characteristics	What characteristics were reported about the interviewer/facilitator? eg, Bias, assumptions, reasons and interests in the research topic	Reasons and interests in the research topic
Domain 2: study design
Theoretical framework
9.	Methodological orientation and Theory	What methodological orientation was stated to underpin the study? eg, grounded theory, discourse analysis, ethnography, phenomenology, content analysis	Phenomenology
Participant selection
10.	Sampling	How were participants selected? eg, purposive, convenience, consecutive, snowball	Purposive
11.	Method of approach	How were participants approached? eg, face-to-face, telephone, mail, email	Face-to-face
12.	Sample size	How many participants were in the study?	18
13.	Non-participation	How many people refused to participate or dropped out? Reasons?	0
Setting
14.	Setting of data collection	Where was the data collected? eg, home, clinic, workplace	A tertiary A hospital

**Table:** 

15.	Presence of non-participants	Was anyone else present besides the participants and researchers?	No
16.	Description of sample	What are the important characteristics of the sample? eg, demographic data, date	Demographic data
Data collection
17.	Interview guide	Were questions, prompts, guides provided by the authors? Was it pilot tested?	Yes
18.	Repeat interviews	Were repeat interviews carried out? If yes, how many?	No
19.	Audio/visual recording	Did the research use audio or visual recording to collect the data?	Yes
20.	Field notes	Were field notes made during and/or after the interview or focus group?	Yes
21.	Duration	What was the duration of the interviews or focus group?	Approximately 30–45 min
22.	Data saturation	Was data saturation discussed?	Yes
23.	Transcripts returned	Were transcripts returned to participants for comment and/or correction?	Yes
Domain 3: analysis and findings
Data analysis
24.	Number of data coders	How many data coders coded the data?	Two
25.	Description of the coding tree	Did authors provide a description of the coding tree?	Yes
26.	Derivation of themes	Were themes identified in advance or derived from the data?	Yes
27.	Software	What software, if applicable, was used to manage the data?	NVivo 12
28.	Participant checking	Did participants provide feedback on the findings?	Yes
Reporting
29.	Quotations presented	Were participant quotations presented to illustrate the themes /findings? Was each quotation identified? eg, participant number	Yes
30.	Data and findings consistent	Was there consistency between the data presented and the findings?	Yes
31.	Clarity of major themes	Were major themes clearly presented in the findings?	Yes
32.	Clarity of minor themes	Is there a description of diverse cases or discussion of minor themes?	Yes

